# Correspondence—Domestic

**Published:** 1885-05

**Authors:** 


### CORRESPONDENCE--DOMESTIC.

"DR. WARREN'S ADVICE."

An interesting relic has been recently found, in the form of a piece of paper containing advice to a patient, in the handwriting of Warren, who fell at Bunker Hill, and whose name is engraved on a stone to be found near the base of the historic shaft which marks the site of that famous battle. Warren, as every one knows, was a member of the medical profession, but no prescription written by him, has, so far as learned, been yet discovered.

The "advice" to a patient, referred to above, is written on a scrap of coarse paper, six by seven inches in dimensions, the handwriting being in the style commonly employed in the colonial and revolutionary periods of the history of this country, nowhere better illustrated than in the diary and other records written by the hand of General Washington. The paper is yellow with age, cracked in several places where it has been folded, and though it bears no signature, the endorsement clearly establishes the identity of the writer. The chirography has been also carefully compared with the diary and other papers left by Dr. Warren, now to be seen in the old South Church and in the Public Library of Boston. Judge Chamberlain, of that library, having made this comparison, declares the manuscript to be genuine and actually written by the hero of Bunker Hill.

It is endorsed on the reverse in the hand of the writer,

"Dr. Warren's advice," and bears in another part the words, "Mr. Pitts." It reads as follows:

"Mr. Pitts will please to take a teaspoonful of the volatile tincture of guiacum, night and morning, in hot water—and two or three teaspoonfuls of the tincture of the bark, daily at noon, in any convenient vehicle.

"He will please to use a diet moderately generous, using such exercise as he can bear without fatigue, and to keep the body open with occasional doses of tincture of hieropicra, about a tablespoonful at a time, or more if on experience it should be found that this quantity should be insufficient to procure the necessary evacuations.

"The astringent applications to the part affected should be continued for several weeks at least, or till it shall arrive at its former size."

The whole is concluded with one of those intricate flourishes which at that time served even better than either initials or the name in full to indicate the identity of the writer.

This interesting paper is in posession of one of the members of the Chicago Historical Society, whose monograph on the Dearborn family has heretofore served as the text for some editorial comments in these columns upon the physicians of the revolutionary period.

Through the kindness of Mr. Daniel Goodwin, Jr., to whom reference is made, we have been permitted to copy the original. J. N. H.

Chicago.

